# Supramolecular nanomedicines based on host–guest interactions of cyclodextrins

**DOI:** 10.1002/EXP.20210111

**Published:** 2023-05-28

**Authors:** Fan Tong, Yang Zhou, Yanyan Xu, Yuxiu Chen, Natalia Yudintceva, Maxim Shevtsov, Huile Gao

**Affiliations:** ^1^ Key Laboratory of Drug Targeting and Drug Delivery Systems West China School of Pharmacy Sichuan University Chengdu China; ^2^ Institute of Cytology of the Russian Academy of Sciences (RAS) St. Petersburg Russia

**Keywords:** cyclodextrins, host–guest interactions, supramolecular nanomedicines

## Abstract

In the biomedical and pharmaceutical fields, cyclodextrin (CD) is undoubtedly one of the most frequently used macrocyclic compounds as the host molecule because it has good biocompatibility and can increase the solubility, bioavailability, and stability of hydrophobic drug guests. In this review, we generalized the unique properties of CDs, CD‐related supramolecular nanocarriers, supramolecular controlled release systems, and targeting systems based on CDs, and introduced the paradigms of these nanomedicines. In addition, we also discussed the prospects and challenges of CD‐based supramolecular nanomedicines to facilitate the development and clinical translation of these nanomedicines.

## Graphical abstract

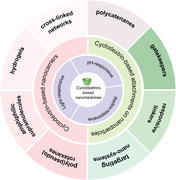

## 1 INTRODUCTION

As we all know, supramolecules usually refer to ordered aggregates formed by two or more building blocks through intermolecular non‐covalent forces, such as hydrophobic interactions, host–guest recognitions, and hydrogen bonding.^[^ ^1^ ^]^ Supramolecular nanotherapeutics have been gradually developed into a compelling approach for tumor diagnosis and therapy.^[^ ^2^ ^]^ Notably, among the benefits of supramolecular nanomaterials are their ideal drug‐loading capacity, high stability, and easy modification.^[^ ^3^ ^]^ Macrocyclic hosts play important roles in supramolecular chemistry.^[^ ^4^ ^]^ Commonly used supramolecular macrocyclic hosts are cyclodextrins (CDs), crown ether, porphyrin, calixarenes, pillararenes,^[^ ^5^ ^]^ cucurbituril,^[^ ^6^ ^]^ and rotaxane.^[^ ^7^ ^]^ Among them, the CDs have attracted the most attention due to their excellent biocompatibility and have been widely applied in biomedical and pharmaceutical applications.^[^ ^8^ ^]^

The CDs were first used as excipients to increase the solubility of hydrophobic medicines and the stability of formulations.^[^ ^9^ ^]^ In recent years, the CDs have been widely exploited as a drug container and building block of nanocarrier to construct supramolecular nanomedicines.^[^ ^10^ ^]^ CDs have a truncated cone structure and consist of a hydrophilic outer surface and a hydrophobic inner cavity,^[^ ^11^ ^]^ having the excellent binding ability with suitable substrates.^[^ ^12^ ^]^ CDs can form inclusion complexes with small molecules,^[^ ^13^ ^]^ peptides, and proteins,^[^ ^14^ ^]^ and self‐assemble into supramolecular nanostructures, exhibiting great potential in drug delivery.^[^ ^15^ ^]^ Additionally, the CDs can not only encapsulate drugs via host–guest interactions to deliver drugs in the form of prodrugs, to decrease toxic effects,^[^ ^16^ ^]^ but also encapsulate drugs, including small molecules,^[^ ^17^ ^]^ peptides, and proteins,^[^ ^18^ ^]^ via formatting nanocarriers based on specific interactions with proper guest molecules to improve their retention in the disease sites.^[^ ^19^ ^]^ Taken together, CDs have been utilized greatly in the field of medicine to improve the physical and chemical properties of parent drugs, such as increasing the solubility and chemical stability of drugs,^[^ ^20^ ^]^ reducing toxicity and side effects,^[^ ^18^, ^21^ ^]^ masking unpleasant odors and tastes.^[^ ^22^ ^]^

Additionally, some functional ligands or groups can be introduced into nanodrugs through CD‐based host–guest interactions, such as targeting ligands and responsive linkers. It is well known that relying on the enhanced permeability and retention effect, the targeting efficiency and release of nanomaterials at tumor sites are still very limited, ascribing to short blood circulation and various biological barriers.^[^ ^23^ ^]^ The introduction of targeting ligands is a viable strategy to improve tumor targeting and specific accumulation in tumors.^[^ ^24^ ^]^ Through these non‐covalent interactions, targeting ligands can be easily introduced into supramolecular nanomedicine to further achieve targeted distribution in tumors.^[^ ^10b^ ^]^ Moreover, CD‐based supramolecules can be designed as “gatekeepers” or “responsive linkers” to achieve controllable release of drugs from nanoparticles.^[^ ^25^ ^]^ Therefore, host–guest interactions of CDs offer innovative strategies for cancer diagnosis and treatment, endowing supramolecular nanomedicines with a high loading capacity of drugs, the controllable release of cargo, and enhanced targeting ability.

**FIGURE 1:**
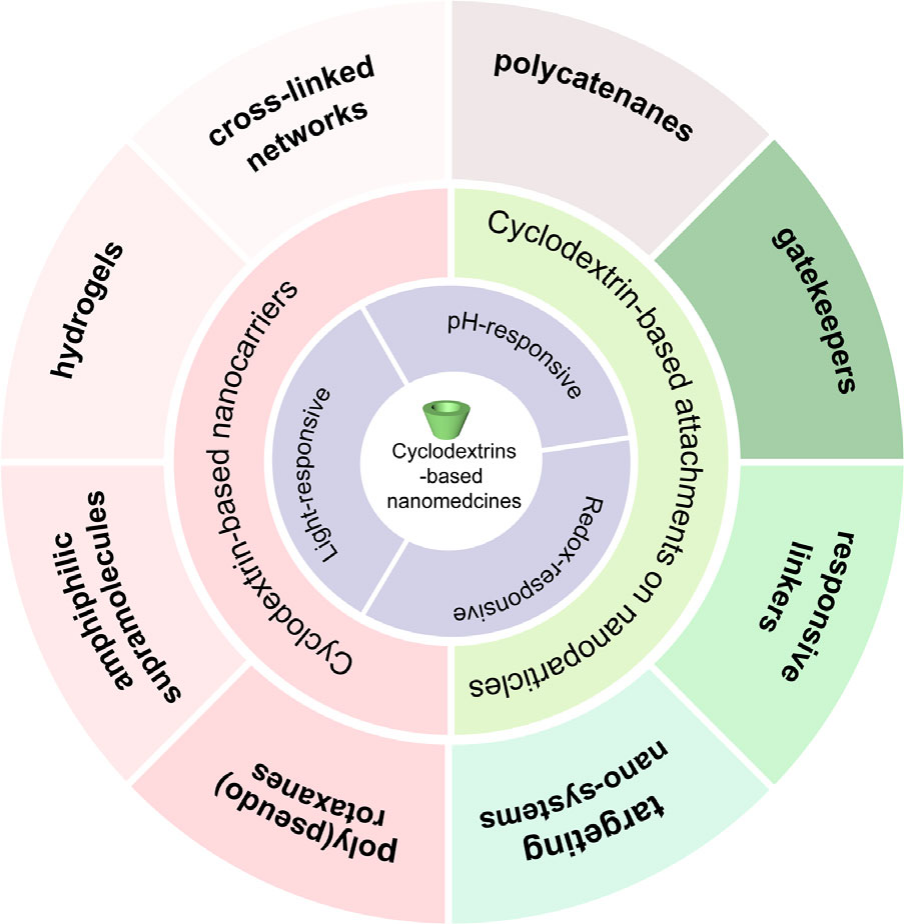
Schematic illustration of cyclodextrin‐based nanomedicines and their outstanding properties.

Herein, we first reviewed the unique properties of CD and its guest molecules, then summarized CD‐based supramolecular nanocarriers, supramolecular controlled release systems, and targeting systems, and introduced the paradigms of these systems (Figure 1 and Table 1). In addition, we also discussed the prospects and challenges of CD‐based supramolecular nanomedicines to promote their development and clinical translation.

**TABLE 1:** Nanomedicines constructed by cyclodextrin‐based host–guest interactions.

CD‐based nanomedicines	Host/guest molecules	Cargoes	Purpose	Cancer cell type	Ref.
Poly(pseudo)rotaxane‐based nanocarriers	α‐CD/PEG	DOX	Targeted delivery and controlled release of DOX	4T1 and NIH3T3 cells	[26]
		MTX	To improve the anticancer activity of MTX	HepG2 cells	[27]
		Ce6, 3BP	Relief of tumor hypoxia to improve PDT efficiency and conversion autophagy from pro‐survival to pro‐death	4T1, HepG2, and KB cells	[28]
		GEM	Enhanced cancer cellular uptake and selective GEM release within cancer cells	BxPC‐3 cells	[29]
		NO, DOX	Mitochondria‐targeted delivery of NO and inhibition of drug resistance and cancer metastasis	MCF‐7 cells	[30]
Amphiphilic supramolecule‐based nanocarriers	β‐CD/BM	DOX	pH‐responsive release of DOX	HepG2 cells	[31]
	β‐CD/PS	PTX	To encapsulate and transfer PTX with a high loading capacity	‐	[32]
	β‐CD/Azo	DOX, SN‐38, phenytoin, and aliskiren	Supramolecular prodrug systems	MDA‐MB‐231 cells	[33]
	β‐CD/Fc	Ce6	Self‐amplified PDT to suppress primary breast cancer and bone metastases	4T1 cells	[34]
	β‐CD/Fc	pDNA	ROS‐responsive release of pDNA	COS‐7 cells	[35]
	β‐CD/Ada	PTX, shRNA	Co‐delivery of chemotherapeutics and siRNAs to suppress cancer growth more effectively	SKOV‐3 cells	[36]
Supramolecular hydrogels	α‐CD/PEG	DOX, ICG, CpG	Abundant tumor‐specific antigen storage in situ and combined immune therapy to inhibit primary tumor growth, tumor recurrence, and metastasis	B16F10 and B16‐OVA cells	[37]
	β‐CD/Ada	‐	Thermo‐responsive hydrogel formation	‐	[38]
	β‐CD/Azo	‐	Photo‐reversible supramolecular hydrogels	‐	[39]
	β‐CD/Fc	GOX, HRP‐H_2_O_2_	Fuel‐driven redox‐responsive hydrogel, a potential glucose sensor	‐	[40]
Supramolecular cross‐linked networks	β‐CD/Ada	CPT, PTX	Reversible control over the size, positron emission tomography (PET) imaging	‐	[41]
	β‐CD/PTX	PTX	Targeted delivery and responsive release of PTX	MCF‐7 cells	[42]
Polycatenane‐based nanocarriers	α‐CD/PEG	‐	Reversible conversion of supramolecule from polyrotaxane to poly(polyrotaxane)	‐	[43]
	β‐CD/PEG	‐	Simple synthesis and separation of polycatenanes	‐	[44]
Supramolecular gatekeepers	β‐CD/Ada	DOX	Active targeting and redox‐responsive drug release	SKOV‐3 cells	[45]
	β‐CD/BM	MXF	pH‐sensitive nanovalve systems	Tularemia	[46]
	β‐CD/Fc	R6G, DOX	pH/redox‐responsive nanovalve, drug size selectivity	‐	[47]
	β‐CD/Azo	FITC	Light‐responsive triggered system	‐	[48]
Supramolecular‐responsive linkers	β‐CD/Ada	JQ1, PPa	Supramolecular prodrug nano‐system, enhance photoimmunotherapy	Panc02 cells	[49]
	β‐CD/Fc	Pt, Ru	PTT, hypoxia relief, photoacoustic, PDT, and computed tomography imaging	4T1 cells	[50]
Supramolecular targeting ligands	β‐CD/Ada	siRNA, Tf	Translation from concept to the clinic for targeted delivery of siRNA	/	[51]
	β‐CD/Ada	BODIPY	Targeted photodynamic killing	MCF‐10A, and MDA‐MB‐231 cells	[52]
	β‐CD/CPT	CPT, RGD, ^64^Cu	Targeted delivery of CPT, PET imaging	4T1 cells	[16b]

## 2 GENERAL PROPERTIES OF CD AND CD‐BASED SUPRAMOLECULES

CD is a water‐soluble macrocyclic oligosaccharide and can be divided into α, β, and γ‐CD (6 to 8 glucose units) according to the number of glucose units.^[^ ^2^, ^12^, ^53^ ^]^ CD is formed by intramolecular glycosylation cyclization of α−1,4‐glycosidic bonds, resulting in a macrocycle with rigid and tapered geometry.^[^ ^54^ ^]^ The conical structure is approximately 7.9 Å in height, and the width widens with an increasing amount of glucose units, and the widths of α, β, γ‐CD are 5.7, 7.8, and 9.5 Å, respectively.^[^ ^55^ ^]^ The outer surface is hydrophilic, benefiting from the hydroxyl groups^[^ ^10^, ^55^, ^56^ ^]^ while the central cavity is lipophilic, due to backbone carbons and ether oxygens of glucose residues.^[^ ^57^ ^]^ Briefly, the CD loop has a rigid conical structure, a hydrophobic core, an electron‐rich entrance, and a hydrophilic outer surface.

In an aqueous solution, the central cavity of CD has a slightly non‐polar nature. And the surrounding water molecules are not conducive to the CD cavity in terms of polar–apolar interactions (energy), which can be spontaneously replaced by suitable low‐polarity object substitution.^[^ ^55a^ ^]^ The driving force for the formation of inclusion complexes is high‐enthalpy water molecules. CD acts as the main part to accommodate the guest molecules, and the two are often associated at a ratio of 1:1. Sometimes there are more complex situations where multiple host/guest molecules participate, such as 2:1, 1:2, and 2:2.^[^ ^9^, ^17^, ^55^ ^]^ The formation of inclusion complexes is mainly affected by the size of the guest molecule or aromatic ring, the position of the hydroxyl group, the presence of hydroxyl and methylene groups on the aliphatic chain, the conformation, and the chirality.^[^ ^55^, ^58^ ^]^ In addition, the complexation is influenced by the solvent used to prepare the complex.^[^ ^59^ ^]^ The hydrophobic cavity of CD can accommodate hydrophobic guest molecules, such as small molecules,^[^ ^17^ ^]^ ions,^[^ ^60^ ^]^ proteins,^[^ ^18^ ^]^ and oligonucleotides,^[^ ^61^ ^]^ thereby improving certain physicochemical properties of the guests, including increasing the solubility and chemical stability of drugs,^[^ ^20^ ^]^ reducing toxicity and side effects,^[^ ^18^, ^21^ ^]^ masking unpleasant odors and tastes,^[^ ^22^ ^]^ protecting drugs from enzymolysis during circulation process,^[^ ^2^, ^62^ ^]^ enhancing drug absorption,^[^ ^56^, ^63^ ^]^ controlling drug release,^[^ ^64^ ^]^ and improving drug permeability across biological barriers.^[^ ^3^, ^65^ ^]^ Moreover, β‐CD has been listed in the “Generally Recognized as Safe” by FDA.^[^ ^56^, ^66^ ^]^ Therefore, CDs have great potential in the fields of biomaterials and biomedicines.

**FIGURE 2:**
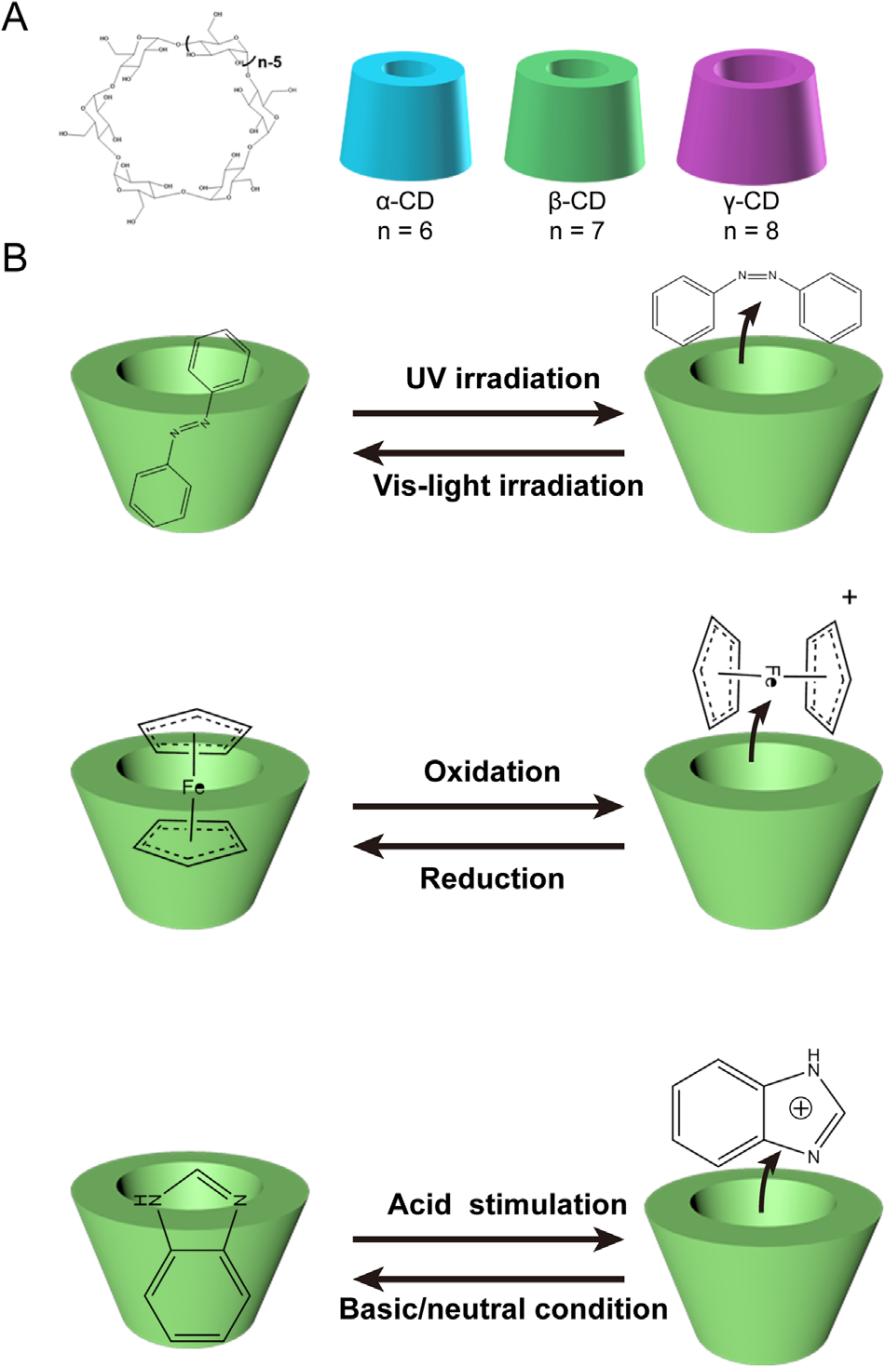
General properties of cyclodextrin (CD) and CD‐based supramolecules. A) Scheme of CDs consists of different numbers of glucose units. B) CD‐based inclusion complexes with different stimuli responsivities.

Meaningfully, the host–guest interactions can serve as the driving force to form nanoparticles. For example, hydrophilic and hydrophobic domains can be introduced through host–guest interactions to form amphiphilic supramolecules, which further self‐assemble to form nanovesicles or micelles.^[^ ^65^, ^67^ ^]^ When specific guest molecules are selected, the supramolecular nanoparticles (SNPs) are further endowed with stimuli responsiveness.^[^ ^15a^ ^]^ Furthermore, when the host or guest molecules are immobilized on the surface of nanoparticles, host–guest interactions or additionally introduced responsive components via host–guest interactions can construct nanodrug delivery systems that respond to various stimuli.^[^ ^25a^ ^]^ Interestingly, the CD can form responsive inclusion complexes with specific guest molecules (Figure 2). This responsive and reversible complex formation/dissociation can be achieved through guest molecules in two distinct states of existence, which can be interconverted using external/internal stimuli.^[^ ^68^ ^]^ And only one form can be encapsulated by the CD cavity. Briefly, there are three main types of such responsive guest molecules, which can respond to light, redox, and pH, respectively.^[^ ^25^, ^58^, ^68^ ^]^

As for the light‐responsive inclusion complex, Azobenzene (Azo) is the typical guest that can form a photo‐sensitive supramolecule with CD. Azo exists in either trans (E) or cis (Z) isomers. Under UV light (∼350 nm) irradiation, trans isomerization is converted to cis, whereas irradiation with visible light (∼450 nm) results in isomerization from cis to trans.^[^ ^58^, ^69^ ^]^ In terms of thermodynamic stability, the trans isomer is more stable, thus achieving cis‐to‐trans conversion via thermal irradiation. The distance between the para carbons of the benzene ring decreases, whereas the dipole moment increases during isomerization from trans to cis.^[^ ^70^ ^]^ Namely, the isomerization of Azo is related to the change of configuration and dipole moment. Non‐polar and linear trans‐Azo readily accesses the hydrophobic cavities of CDs while polar cis isomer does not favor the steric requirements for the formation of host–guest complexes.^[^ ^71^ ^]^ Therefore, Azo has great potential for constructing photo‐switchable supramolecular composite materials.^[^ ^64^, ^72^ ^]^

Given the different redox states and ROS levels in physiological/pathological microenvironments, considerable efforts have been devoted to developing nanomedicines with redox sensitivity. Ferrocene (Fc), another guest molecule, is frequently utilized to fabricate redox‐responsive supramolecular systems based on CD inclusion complexes.^[^ ^64^, ^73^ ^]^ According to the size of the CD cavity, non‐polar Fc enters the hydrophobic cavity of CD in different ways. The binding ratio of Fc to α‐CD is 2:1, while the binding ratio of Fc to β‐CD and γ‐CD is 1:1. The Fc enters the β‐CD lumen axially while being evenly clamped by the larger γ‐CD loop.^[^ ^58^, ^74^ ^]^ When Fc is (electro)chemically oxidized, the Fe^2+^ is oxidized to Fe^3+^, and the neutral hydrophobic Fc is converted into the hydrophilic Fc^+^, which in turn leads to the detachment of Fc^+^ from the hydrophobic cavity and the dissociation of inclusion complex.^[^ ^75^ ^]^ Thus, Fc can be applied to design supramolecular systems with reversible redox responsiveness.

On the other hand, the tumor microenvironment (TME) is slightly more acidic than that of healthy tissues due to the overproduction of acidic metabolites. So pH‐responsive supramolecules have broad application prospects in drug delivery and tumor therapy.^[^ ^76^ ^]^ Benzimidazole (BM) as a basic guest molecule, is unprotonated and uncharged at physiological pH and can form inclusion complexes with CD.^[^ ^15^, ^25^, ^64^ ^]^ At lower pH (<6), BM is protonated and positively charged, causing detachment from the CD cavity.^[^ ^77^ ^]^ Briefly, BM offers CD‐based inclusion complexes pH responsivity.

Therefore, through CD‐based host–guest interactions, various stimuli‐responsive supramolecular systems can be constructed and further utilized for drug delivery. Responsive supramolecular nanomedicines constructed through host–guest interactions will be reviewed in detail in the following sections.

## 3 CYCLODEXTRIN‐BASED SUPRAMOLECULAR NANOCARRIERS

The efficacy of antitumor drugs is often greatly limited by various factors such as poor solubility, instability, drug resistance, non‐specific distribution, and toxicity of drugs.^[^ ^2^, ^19^, ^56^, ^78^ ^]^ Utilizing the EPR effect and active targeting, nanodrugs can specifically distribute to tumor sites to improve antitumor efficacy and reduce side effects.^[^ ^23^, ^79^ ^]^ However, the unsatisfied degradability of nanocarrier hinders its clinical translation.^[^ ^80^ ^]^ There are non‐negligible problems of exogenous carriers such as poor metabolism, low clearance rate, and adverse interactions with various components of the immune system, which inevitably lead to adverse reactions and immunotoxicity.^[^ ^16^, ^81^ ^]^ Thus, there is an urgent need to construct novel nanoplatforms to deliver drugs to tumors efficiently, with rapid degradation ability. Recently, CD‐based host–guest systems have been widely utilized as delivery vehicles and drug containers to equip nanomedicines with great biocompatibility and degradability.^[^ ^19^, ^64^, ^82^ ^]^

The high selectivity of host–guest interactions offers great possibilities for the construction of structurally diverse and programmable functional supramolecular biomaterials.^[^ ^83^ ^]^ The preparation process of host–guest inclusion complexes is simple and reversible, and it is possible to construct supramolecular systems that respond to different external/internal stimuli.^[^ ^7^, ^68^, ^84^ ^]^ In addition, the uniqueness of the molecule‐level regulation of self‐assembled components further allows for flexible design and controllability of synthetic supramolecular materials with desirable size and morphological changes to suit specific applications.^[^ ^84^ ^]^ In this section, according to structure and composition, SNPs are classified into rotaxanes and poly(pseudo)rotaxanes, amphiphilic supramolecules, cross‐linked hydrogels and networks, and polycatenanes.

### 3.1 Rotaxane and poly(pseudo)rotaxane‐based nanocarriers

Rotaxanes are mechanically interlocked molecular structures in which cyclic molecules are strung into a chain‐like structure, and a bulky terminator is conjugated to the end of the chain axis to prevent the dissociation of the cyclic molecules.^[^ ^55^, ^85^ ^]^ The necessary step in rotaxane formation is the threading of the chain shaft. Rotaxane monomers are polymerized by covalent linkage to obtain polyrotaxanes.^[^ ^86^ ^]^ Poly(pseudo)rotaxanes are supramolecular threads governed by reversible and dynamic interactions between CDs and polymers, in which multiple CD loops are driven by inclusion complexation, trapped on the polymer chain.^[^ ^87^ ^]^ Suitable polymer chains for forming poly(pseudo) rotaxanes include liner polymers (homopolymers and block copolymers) and branched polymers (grafts and star polymers).^[^ ^75^, ^85^ ^]^ There are various guest homopolymers for constructing poly(pseudo)rotaxane systems, such as polyethylene glycol (PEG),^[^ ^88^ ^]^ polyethylene oxide (PEO),^[^ ^89^ ^]^ poly(ε)‐caprolactone (PCL),^[^ ^85a^ ^]^ and poly(propylene oxide) (PPO).^[^ ^90^ ^]^ Among them, α‐CD can form inclusion complexes with PEG or PCL, but not with PPO because of the small hydrophobic cavity. β‐CD, with a larger cavity, can interact with PCL or PPO but not PEG. γ‐CD has the largest cavity, so PPO and even two PEG/PCL chains can penetrate.^[^ ^15^, ^75^, ^85^, ^91^ ^]^ Notably, the most widely studied poly(pseudo)rotaxane system is α‐CD/PEG. Therefore, in this part, we are supposed to introduce the CD/PEG‐based poly(pseudo)rotaxane systems for drug delivery.

During the formation process of supramolecules, drugs can be encapsulated into poly(pseudo)rotaxane systems. Liu et al. chose cholic acid as the capping group to prepare α‐CD/PEG poly(pseudo)rotaxane and encapsulate the chemotherapeutic drug doxorubicin (DOX) during the self‐assembly.^[^ ^26^ ^]^ Cholic acid is naturally compatible with the glucosamine unit, which can recognize some cancer cells such as 4T1 cells that highly express glucose transporters,^[^ ^92^ ^]^ endowing the poly(pseudo)rotaxane with active targeting capacity. Gu and co‐workers prepared α‐CD/PEG SNPs to deliver methotrexate (MTX), an anti‐folate antitumor drug.^[^ ^27^ ^]^ In this study, the UV absorption peaks of MTX after encapsulation showed a red‐shift, indicating a strong interaction with SNPs. During the encapsulation process, MTX disrupted the poly(pseudo)rotaxane crystal structure, resulting in the SNPs changing from a regular spherical to a spindle shape. In addition, MTX can be rapidly released from poly(pseudo)rotaxane nanoparticles, showing anticancer activity superior to that of the free drug.^[^ ^27^ ^]^ Thus, hydrophobic drugs can be physically or electrostatically encapsulated into SNPs during poly(pseudo)rotaxane formation, which significantly improves the bioavailability and reduces the non‐specific distribution and toxicity of drugs.

**FIGURE 3:**
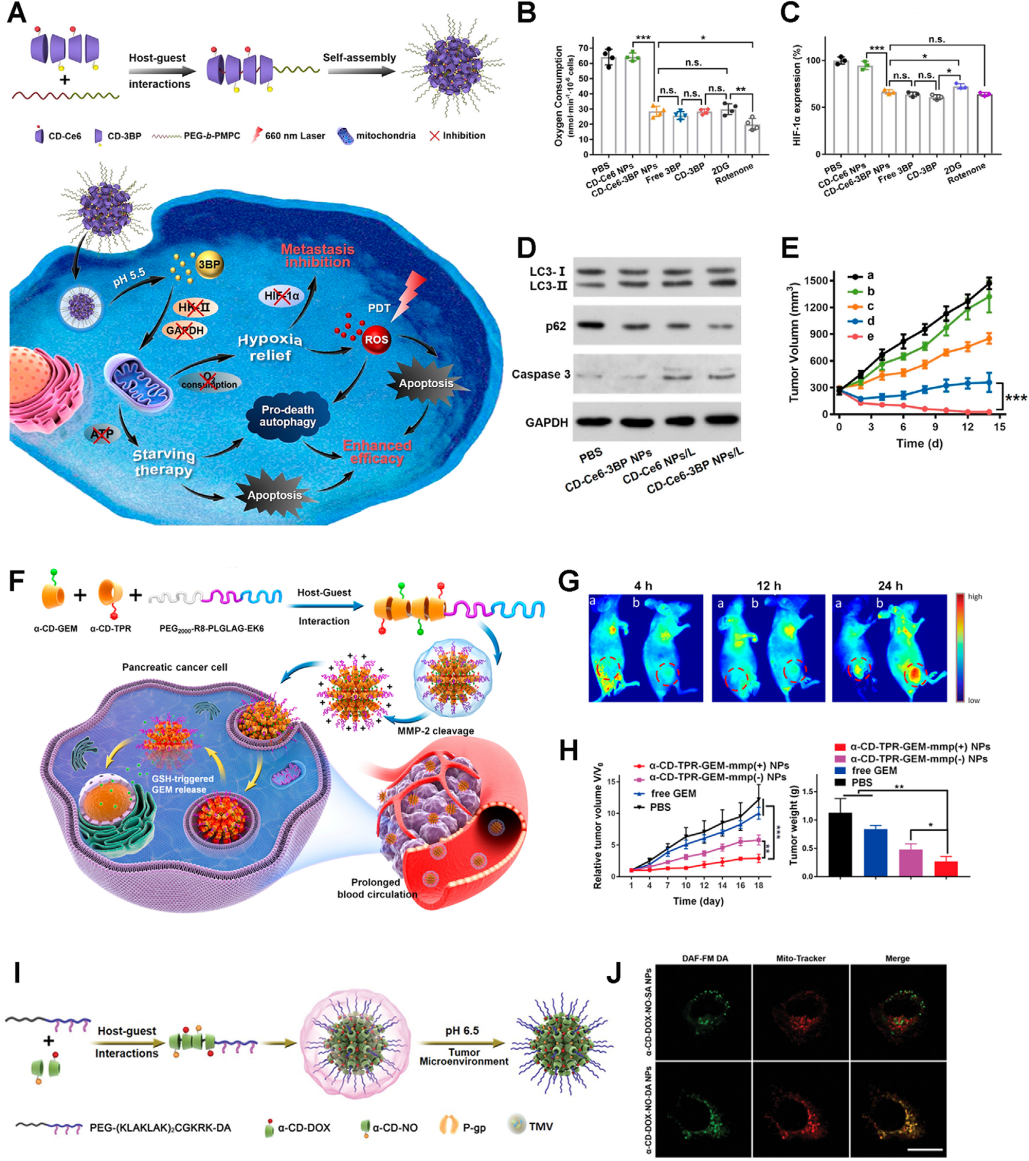
Poly(pseudo)rotaxane‐based nanocarriers. A) Design and preparation schematics of CD‐3BP‐Ce6 NPs to improve the efficiency of photodynamic therapy. B) Oxygen consumption and C) HIF‐1α expression analysis of KB cells. D) Investigation of autophagy and apoptosis levels in tumors. E) Antitumor effect of CD‐3BP‐Ce6 NPs in vivo. Reproduced with permission.^[^ ^28^ ^]^ Copyright 2020, American Chemical Society. F) Fabrication and antitumor mechanism schematics of TPR. G) Biodistribution of α‐CD‐TPR‐GEM‐MMP (−) NPs (a) and α‐CD‐TPR‐GEM‐MMP (+) NPs (b), respectively. H) Antitumor effect of different formulations. Reproduced with permission.^[^ ^29^ ^]^ Copyright 2020, American Chemical Society. I) Synthesis and fabrication of α‐CD‐DOX‐NO‐DA NPs. J) Mitochondria targeting assay of α‐CD‐DOX‐NO‐DA NPs. Reproduced with permission.^[^ ^30^ ^]^ Copyright 2020, Wiley‐VCH.

The release of the above‐mentioned drugs from SNPs depended on the dissociation of poly(pseudo)rotaxanes, which is uncontrollable and brings undesirable side effects. To achieve a controlled release of drugs and reduce side effects, drugs can also be bound to poly(pseudo)rotaxane nanoparticles via labile covalent bonds.^[^ ^56^, ^93^ ^]^ Ji group used the α‐CD/PEG poly(pseudo)rotaxane supramolecules to construct a series of nanodrugs for tumor chemotherapy, imaging, photodynamic therapy (PDT), gas therapy, and combination therapy (Figure 3).^[^ ^28^, ^29^, ^30^ ^]^ They investigated the effect of the α‐CD/PEG poly(pseudo)rotaxane system to sensitize PDT of breast cancer (Figure 3A).^[^ ^28^ ^]^ The respiratory inhibitor 3‐bromopyruvate (3BP) was conjugated to α‐CD (CD‐3BP) via a pH‐sensitive hydrazone bond, synergizing with the photosensitizer chlorin e6 (Ce6)‐linked α‐CD, and interacted with polyethylene glycol‐b‐poly(2‐methacryloyloxyethylphosphorylcholine) (PEG‐b‐PMPC) to form poly(pseudo)rotaxanes and further self‐assembled to supramolecular co‐delivery nanoplatforms. 3BP was released in response to acid and reduced oxygen consumption by inhibiting respiration, thereby alleviating hypoxia, enhancing PDT benefit, and leading to starvation‐induced autophagy, which synergized with ROS‐induced autophagy to trigger excessive autophagy, which in turn promoted apoptosis (Figure 3B–E). Therefore, drugs can be bound to SNPs via unstable covalent bonds, endowing supramolecular nanomedicines with the controllable or stimuli‐responsive release.

Notably, introducing targeting ligands will further control the responsive release of drugs at specific sites. For example, α‐CD‐TPR and α‐CD‐GEM were obtained by conjugating aggregation‐induced emission (AIE) active molecule TPR and redox‐sensitive gemcitabine (GEM) prodrug with α‐CD, respectively. The poly(pseudo)rotaxane system was formed via interactions between α‐CD‐TPR/α‐CD‐GEM and MMP‐2 cleavable peptide‐modified PEG (PEG2000‐R_8_‐PLGLAG‐EK_6_) (Figure 3F).^[^ ^29^ ^]^ The cell‐penetrating peptide R8 and the zwitterionic stealth sequence EK_6_ endowed nanomedicine with cell‐penetrating and “stealth” abilities.^[^ ^29^ ^]^ After accumulation in the tumor and uptake by cancer cells, the release of GEM was induced by high levels of intracellular GSH, showing an excellent antitumor effect (Figure 3G,H). Similarly, PEG was modified with dimethyl maleic anhydride (DA), as an acid cleavable group to bind PEGylated mitochondrial internalizing peptide (PEG‐(KLAKLAK)_2_CGKK) (Figure 3I). DOX and NO‐modified α‐CD self‐assembled with PEG‐(KLAKLAK)_2_CGKK to form acid‐activated charge‐reversal nanomedicines for delivering NO to mitochondria precisely to relieve drug resistance and suppress metastasis of breast cancer (Figure 3J).^[^ ^30^ ^]^ Briefly, targeting ligands and drugs can be introduced into poly(pseudo)rotaxane‐based SNPs via responsive bonds, allowing drugs release at specific sites.

Additionally, modification of the ribbon polymer using a sensitive capping mechanism enables the triggering of drug release upon removal of the end cap modification.^[^ ^94^ ^]^ Hideyoshi et al. developed a nonviral gene carrier based on a poly(pseudo)rotaxane, consisting of many cationic α‐cyclodextrins (α‐CDs) and a disulfide‐introduced PEG.^[^ ^94^ ^]^ When disulfide bonds were cleaved, the non‐covalent linkages between α‐CDs and PEG dissociated, leading to the release of pDNA. In brief, poly(pseudo)rotaxane‐based SNPs have great application prospects in drug delivery and controlled drug release, which encapsulate drugs or conjugate drugs via unstable covalent bonds.

### 3.2 Amphiphilic supramolecule‐based nanocarriers

Amphiphilic supramolecules combining hydrophilic domains with hydrophobic domains by host–guest interaction can self‐assemble to form nanostructures for drug encapsulation, similar to liposomes or micelles.^[^ ^67^, ^95^ ^]^ Amphiphilic supramolecules are usually formed by the complexation of guest‐containing hydrophobic units with host‐containing hydrophilic units, many of which dissociate in response to external stimuli for drug release or imaging purposes.^[^ ^68a^ ^]^ Amphiphilic supramolecules are also rendered stimuli responsive when specific guest molecules are introduced, such as BM, Azo, and Fc. Moreover, after rational design, amphiphilic supramolecules can be used to construct smart nanoplatforms to co‐deliver different drugs.

**FIGURE 4:**
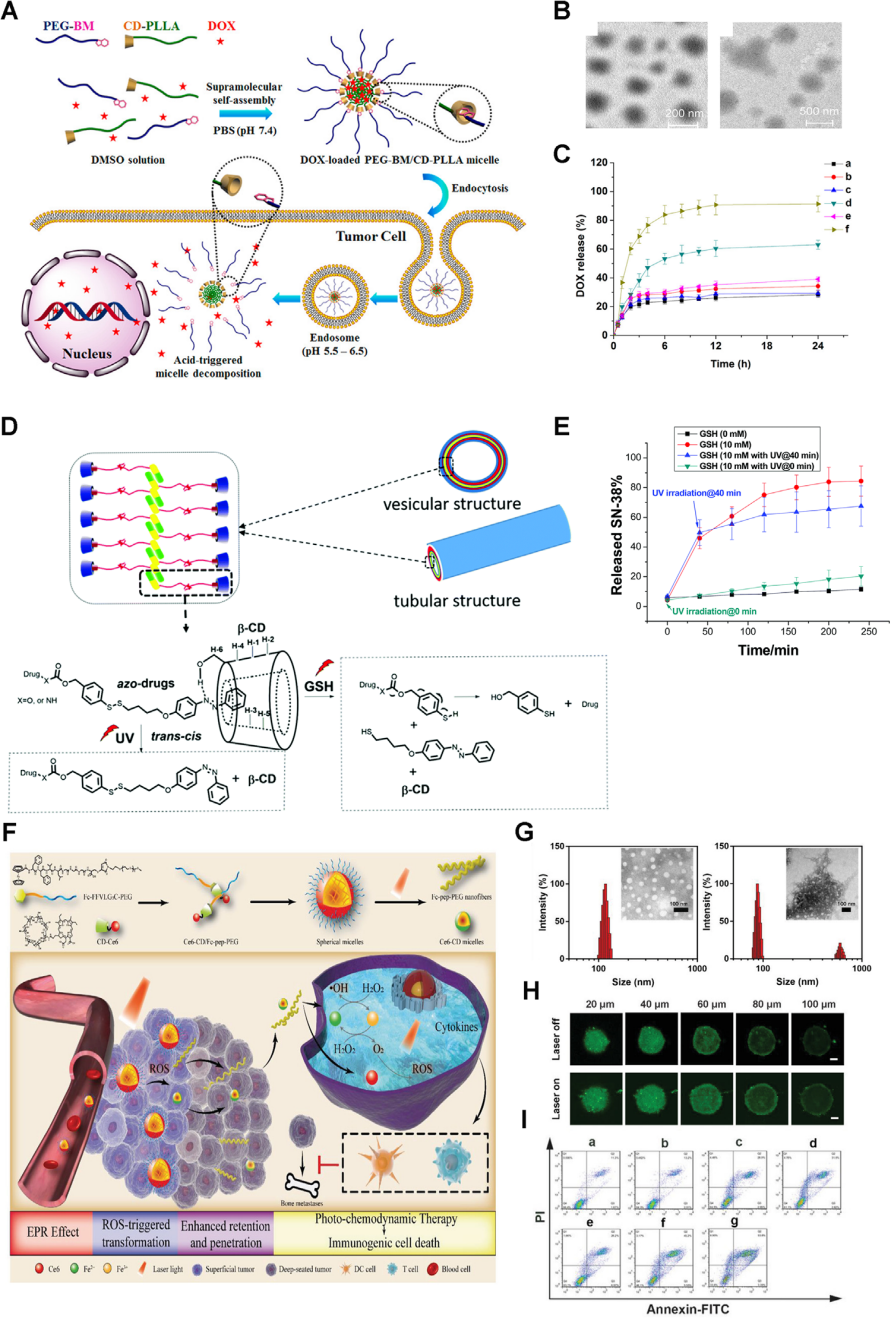
Amphiphilic supramolecule‐based nanocarriers. A) Preparation and responsive cargo release schematics of DOX‐loaded PEG‐BM/CD‐PLLA. B) TEM images of PEG‐BM/CD‐PLLA at different pH (left: 7.4, right: 5.5). C) Release profiles of DOX in a medium with various pH values. Reproduced with permission.^[^ ^31^ ^]^ Copyright 2015, American Chemical Society. D) Preparation and dual‐responsive release schemes of β‐CD/azo‐SN‐38. E) Release profiles of β‐CD/azo‐SN‐38 under different conditions. Reproduced with permission.^[^ ^33^ ^]^ Copyright 2017, Royal Society of Chemistry. F) Fabrication and combination therapy schematics of Ce6‐CD/Fc‐pep‐PEG. G) TEM micrographs of Ce6‐CD/Fc‐pep‐PEG without (left) and with (right) 650 nm laser irradiation. H) The penetration and retention of Ce6‐CD/Fc‐pep‐PEG in vitro. I) Antitumor effect of different formulations in vitro. Reproduced with permission.^[^ ^34^ ^]^ Copyright 2021, Wiley‐VCH.

As mentioned above, the TME exhibits a lower pH value than normal tissues, and pH‐sensitive nanomedicines have great prospects in the imaging and therapy of cancer. Amphiphilic supramolecules with pH responsivity are easily developed by combining hydrophilic and hydrophobic domains based on CD/BM interactions. Chen's group developed a series of β‐CD‐based pH‐sensitive drug delivery systems.^[^ ^31^, ^96^ ^]^ Typically, they constructed supramolecular micelles with pH‐responsivity based on BM‐modified PEG (PEG‐BM) and β‐CD‐grafted poly(l‐lactide) (CD‐PLLA), and encapsulated model drug DOX into micelles (Figure 4A).^[^ ^31^ ^]^ The BM was protonated when the pH decreased, causing the micelles to expand. As expected, the release of DOX was significantly accelerated once the pH changed to 5.5 (Figure 4B,C). Similarly, Yazdi and co‐workers also reported pH‐responsive vesicles to deliver hydrophobic drugs such as paclitaxel (PTX) with high loading capacity.^[^ ^32^ ^]^ In this supramolecular vesicle, β‐CD‐linked polyglycerol (β‐CD‐HPG) was used as the host, and polystyrene was used as the guest. Since the above supramolecular nanoplatforms are constructed through CD/BM‐based host–guest interactions, lowering of pH leads to the dissociation of the building domains and release of the drug, which is beneficial for efficient therapy of cancer.

Similarly, by combining hydrophilic and hydrophobic components through CD/Azo interactions, light‐responsive amphiphilic supramolecules can be easily synthesized and designed to construct drug delivery systems. Yan et al. constructed Janus hyperbranched polymer (JHBP)‐based light‐controlled vesicles via CD/Azo host–guest interactions.^[^ ^97^ ^]^ These carriers consisted of Azo‐modified hyperbranched poly(3‐ethyl‐3‐oxetanecarbinol) (Azo‐g‐HBPO) and hydrophilic β‐CD‐capped hyperbranched polyglycerol (CD‐g‐HPG) composition. Jiang group reported supramolecular amphiphiles based on interactions between CDs and Azo‐labeled hydrophobic drugs, such as Azo‐modified 7‐ethyl‐10‐hydroxycamptothecin, DOX, phenytoin, and aliskiren (Figure 4D).^[^ ^33^ ^]^ Under UV irradiation, the trans‐Azo group isomerized to cis‐Azo, causing the reversible dissociation of amphiphilic supramolecular vesicles and drugs released from vesicles(Figure 4E). Thus, these amphiphilic supramolecules are excellent candidates for hydrophobic drug carriers, which are equipped with light responsivity.

Aimed at excessive ROS concentration in the TME, researchers have developed a great number of smart nanoplatforms with redox responsiveness to deliver various drugs.^[^ ^34^, ^98^ ^]^ Qin and co‐workers reported self‐delivered supramolecular nanomedicine (Ce6‐CD/Fc‐pep‐PEG) based on chlorin e6‐conjugated β‐CD and Fc‐FFVLG_3_C‐PEG linkages (Figure 4F–I).^[^ ^34^ ^]^ Upon reaching the tumor, hydrophobic Fc turned into hydrophilic Fc^+^ once oxidized by overexpressed ROS in the TME, thus leading to the weakened hydrophobic host–guest interaction and the destruction of the inclusion complex. Notably, driven by photosensitizer Ce6, constant generation of ROS supplemented its insufficiency and uneven distribution in the tumor region, as well as guaranteed the continuous occurrence of Fenton reaction catalyzed by chemodynamic therapy agent Fc, which eventually relieved hypoxia and amplified PDT efficiency (Figure 4F,I). Meanwhile, owing to the intermolecular hydrogen bonds, the Fc‐pep‐PEG reconstructed to form nanofibers with enhanced retention ability, while contractible Ce6‐CD retained the spherical shape with better penetration (Figure 4G,H). Similarly, Yuan et al. studied amphiphilic PEG‐b‐PS copolymers via the recognition interaction between β‐CD‐terminated polystyrene (PS‐β‐CD) and Fc‐PEG.^[^ ^99^ ^]^ Briefly, under alternating redox conditions, supramolecular β‐CD/Fc inclusion complexes reversibly associated and dissociated, enabling drugs to load into and release from amphiphilic supramolecular nanocarriers.

In addition, host–guest recognition has also been widely applied to build smart nanoplatforms to achieve co‐delivery of drugs/prodrugs and genes, resulting in synergistic anticancer effects after the responsive release of drugs and nucleotides. Tang and colleagues reported a smart supramolecular gene carrier, polyethyleneimine‐modified β‐CD (PEI‐β‐CD).^[^ ^36^, ^100^ ^]^ For example, amantadine (Ada)‐modified PTX could self‐assemble with PEI‐β‐CD and short hairpin RNA (shRNA) to form nanoparticles.^[^ ^36^ ^]^ Some researchers used the recognition effect of CD/Fc to construct a smart supramolecular gene carrier, which integrates the benefits of traditional polymers and supramolecules, with high stability, excellent biological properties, degradability, and intelligent responsiveness.^[^ ^35^ ^]^ Supramolecular polymerization was achieved by host–guest complexation of PEG‐CD and Fc‐PEHA‐CD. The supramolecular copolymer dissociated and the condensed pDNA could be completely released once Fc was oxidized by H_2_O_2_ to Fc^+^.^[^ ^35^ ^]^ In brief, through ingenious design, amphiphilic supramolecules can not only achieve multi‐drug co‐loading or gene/drug co‐delivery but also controllable drug release in response to different stimuli.

### 3.3 Supramolecular hydrogels and cross‐linked networks

From the topological point of view, CD‐based supramolecular gels are divided into (1) CD‐based poly(pseudo)rotaxane supramolecular hydrogels; (2) hydrogels formed by cross‐linking of CDs and small molecule guests.^[^ ^75^, ^101^ ^]^ Poly(pseudo)rotaxane‐based hydrogels consist of CD with a cavity structure and appropriate polymers’ physicochemical properties that form necklace‐like supramolecules.^[^ ^102^ ^]^ Among them, α‐CD/PEG is the most common. The formation mechanism of the gel may be that in aqueous solution terminals of PEG can string into the inner cavity of α‐CD. The α‐CD loops penetrating the PEG can be hydrogen‐bonded to form poly(pseudo)rotaxanes. α‐CD becomes hydrophobic with hydroxyl groups depleting, and α‐CD/PEG complexes aggregate and act as a cross‐linker, thereby triggering supramolecular hydrogel formation.^[^ ^88^, ^103^ ^]^ Moreover, hydrogels are thixotropic and reversible.^[^ ^104^ ^]^ The viscosity of the hydrogel drops sharply when a shearing force is applied and returns to its previous viscosity within a short time when the shearing force is removed.^[^ ^84^ ^]^ Therefore, α‐CD/PEG supramolecular hydrogels serve as potential drug delivery materials and can be utilized as injectable systems for antitumor therapy.

**FIGURE 5:**
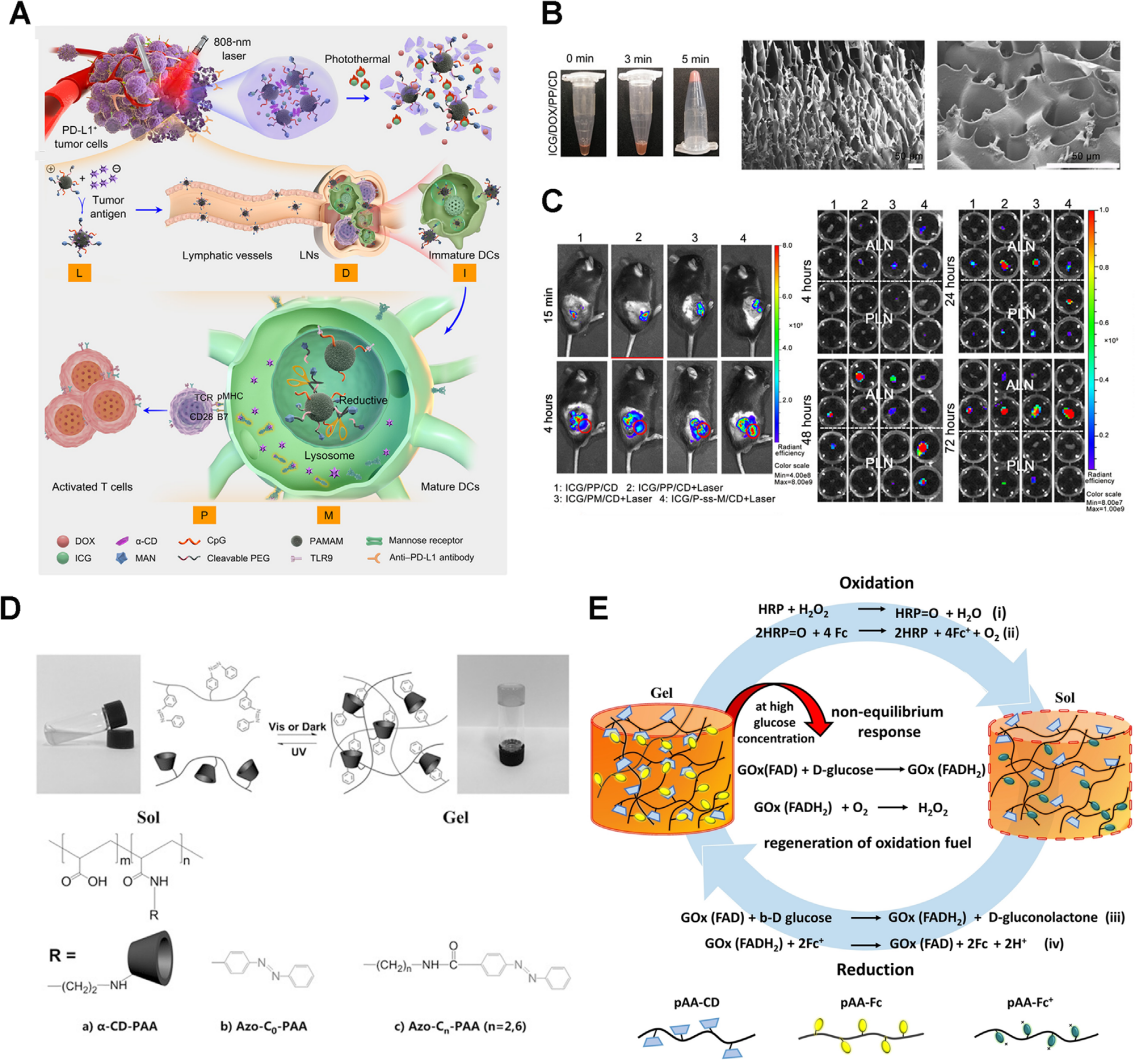
Supramolecular hydrogels based on cyclodextrin (CDs). A) Scheme illustration of CpG‐P‐ss‐M‐mediated LDIMP process. B) Photograph of the gelation process after ultrasonication and SEM micrograph of PP/CD gel. C) Biodistribution of indocyanine green‐loaded hydrogels in tumor‐bearing mice and ex vivo lymph nodes. Reproduced with permission.^[^ ^37^ ^]^ Copyright 2020, AAAS. D) Schematics of reversible sol–gel transition of α‐CD‐PAA/Azo‐Cn‐PAA polymer network. Reproduced with permission.^[^ ^39^ ^]^ Copyright 2016, Wiley‐VCH. E) Schematic illustration of fuel‐driven redox‐responsive hydrogel comprised of pAA‐CD and pAA‐Fc. Reproduced with permission.^[^ ^40^ ^]^ Copyright 2021, Wiley‐VCH.

Drugs can be loaded into α‐CD/PEG supramolecular hydrogels in situ at room temperature. The hydrogel administrated into the tumor acts as a drug depot, releasing drugs continuously and exerting a therapeutic effect. Gao et al. reported a targeting multifunctional hydrogel system for combined treatment of melanoma recurrence and metastasis (Figure 5A). Among them, PAMAM, decorated with mannose‐linked GSH‐responsive PEG and immunomodulator CpG (CpG‐P‐ss‐M), was the main part of gel formation, which realized the programmable tumor and lymph nodes targeting (Figure 5B).^[^ ^37^ ^]^ The chemotherapeutic drug DOX and indocyanine green (ICG), a photothermal agent, were encapsulated into the hydrogel at room temperature. Under NIR irradiation, DOX was released, synergizing with PTT to induce potent ICD. Simultaneously released CpG‐P‐ss‐M drained to lymph nodes and induced DC maturation, thereby effectively combining chemotherapy, PTT, and immunotherapy (Figure 5C). In addition to in situ encapsulation, drugs can also be loaded by forming conjugates with poly(pseudo)rotaxane. Nobuhiko Yui et al. constructed poly(pseudo)rotaxane by passing the α‐CD ring along an l‐phenylalanine (l‐Phe)‐terminated PEG chain.^[^ ^105^ ^]^ Theophylline was specifically bound to α‐CD and thus theophylline‐poly(pseudo)rotaxane conjugates formed spontaneously. After the degradation of the poly(pseudo)rotaxane terminal peptide group, the combination of theophylline and α‐CD was hydrolyzed, and active drugs were gradually released. Therefore, drugs can be loaded into poly(pseudo)rotaxane hydrogels in situ or by forming conjugates, which act as drug storages to release drugs continuously.

Besides polymer chains, small molecular guests can also interact with CD cavities under aqueous conditions, acting as cross‐linkers for the formation of supramolecular hydrogels. The size matching and environmental conditions (such as temperature, light, redox, and pH) play a role in the association/dissociation of inclusion complexes.^[^ ^68c^ ^]^ Environmental changes cause the association/dissociation of supramolecules, endowing supramolecules with responsiveness to various stimuli. Thus, adamantane (Ada), Fc (redox responsive), Azo (light responsive), and BM (pH responsive) are potential guest molecules of CDs to form supramolecular hydrogels.^[^ ^75^, ^106^ ^]^

β‐CD/Ada supramolecular hydrogels have broad application prospects due to their self‐healing, shape memory, and injectable properties.^[^ ^38^, ^107^ ^]^ Li and co‐workers prepared a star supramolecular structure by the inclusion of the star polymer PNIPAAm (low critical temperature of 32°C) with a β‐CD core and Ada‐terminated 8‐arm PEG. The subsequent aggregation formed 3D networks, namely, thermally responsive and reversible smart hydrogels.^[^ ^38^ ^]^ Photo‐sensitive hydrogels have also caught the attention of scientists.^[^ ^39^, ^108^ ^]^ Guo and co‐workers conjugated α‐CD and Azo onto polyacrylic acid (PAA) chains, respectively.^[^ ^39^ ^]^ Then, photo‐sensitive α‐CD‐PAA/Azo‐PAA hydrogels were prepared by encapsulating Azo into CD (Figure 5D). When concentrations of α‐CD‐PAA and Azo‐PAA, degree of substitution, and length of chain increased, the shear viscosity of hydrogels increased.^[^ ^39^ ^]^ Researchers also constructed redox‐responsive hydrogels based on CD/Fc inclusion complex.^[^ ^40^, ^109^ ^]^ Bart Jan and co‐workers reported hydrogels with β‐CD/Fc inclusion complex as supramolecular cross‐linkers. the redox‐responsivity was triggered by the horse radish peroxidase (HRP)–H_2_O_2_ and glucose oxidase (GOx)–d‐glucose (Figure 5E).^[^ ^40^ ^]^ Additionally, Akira and co‐workers linked β‐CD and Fc to PAA side chains, respectively, to obtain the β‐CD‐PAA host polymer and Fc‐PAA guest polymer.^[^ ^109a^ ^]^ The two formed a host–guest polymer hydrogel with redox‐responsive and self‐healing abilities. Moreover, there are other guest molecules utilized to prepare hydrogels, including cholesterol,^[^ ^110^ ^]^ BM,^[^ ^111^ ^]^ bile acid,^[^ ^112^ ^]^ tert‐butyl,^[^ ^113^ ^]^ phenolphthalein, bipyridine, and dansyl.^[^ ^75a^ ^]^ In brief, by choosing suitable small guest molecules, the construction of supramolecular gels is easy to achieve, and responds to various stimuli.

**FIGURE 6:**
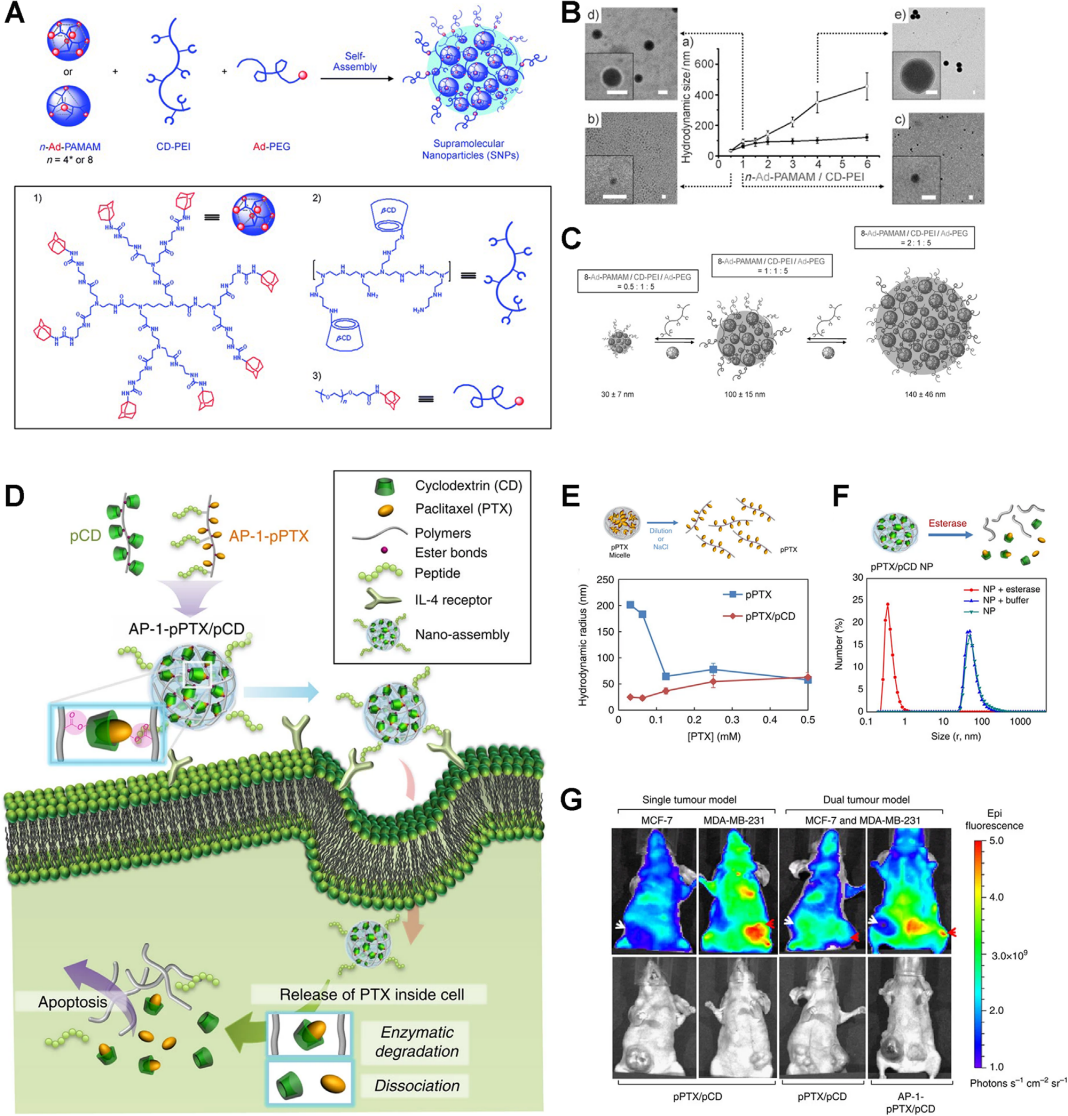
Supramolecular networks based on cyclodextrins. A) Modular synthesis and preparation of supramolecular nanoparticles (SNPs) with controlled size. B) TEM images and particle sizes when ratios of AD‐PAMAM/CD‐PEI increase. C) Controllable sizes of SNPs. Reproduced with permission.^[^ ^41^ ^]^ Copyright 2009, Wiley‐VCH. D) Schematics of nano‐assembly‐mediated PTX delivery. E) Illustration of pPTX micelle disruption and the relationship between sizes and PTX concentration of pPTX/pCD and pPTX micelles. F) Enzymatic degradation and size change of pPTX/pCD and pPTX. G) In vivo biodistribution of pPTX/pCD micelles. Reproduced with permission.^[^ ^42^ ^]^ Copyright 2014, Springer Nature.

Moreover, the supramolecule backbone can also be cross‐linked by covalently linked guest molecules and form a supramolecular nanoplatform with a cross‐linked network structure instead of hydrogels. Self‐assembly of cross‐linked network SNPs for drug delivery used specific recognition between CDs and guest molecules.^[^ ^41^, ^114^ ^]^ Tseng et al. used amantadine (AD)‐conjugated polyamide dendrimer (AD‐PAMAM), PEG (AD‐PEG), and β‐CD‐linked polyethyleneimine (CD‐PEI) to prepare cross‐linked networks of variable size (Figure 6A).^[^ ^41^ ^]^ Adjusting the ratio of the three, controllable SNPs with diverse particle sizes (30–450 nm) were successfully constructed (Figure 6B,C). Researchers also developed SNPs benefiting from interchain and intrachain supramolecular cross‐linking based on the specific interaction between CDs and chemotherapeutic agents such as CPT and PTX.^[^ ^42^, ^82^, ^115^ ^]^ Won Jong group prepared a nanomedicine based on polymeric CD (pCD) and polymeric PTX (pPTX) (Figure 6D).^[^ ^42^ ^]^ CD and PTX were grafted to maleic anhydride copolymers via breakable ester bonds, respectively, and the IL‐4 receptor targeting peptide AP‐1 was further introduced, endowing the supramolecular nanomedicine with high loading capacity, extended circulation time, tumor targeting delivery, and controllable drug release in tumor cells (Figure 6E–G). Similarly, supramolecules can be cross‐linked by specific guest molecules, and further self‐assemble to form a cross‐linked network with nano‐size. Therefore, through host–guest recognitions, supramolecular cross‐linked networks can be constructed, which further assemble into hydrogels or nanoparticles.

### 3.4 Polycatenane‐based nanocarriers

Polycatenanes are also mechanically interlocked molecules that can be divided into main chain, side chain, linking, radial, and network types.^[^ ^116^ ^]^ Unfortunately, the synthesis of corresponding alkanes and polyalkanes is very difficult, compared to CD‐based rotaxanes and poly(pseudo)rotaxanes, among which the synthesis of main‐chain polyalkanes with linear structure is the most challenging.^[^ ^116^, ^117^ ^]^ The synthesis and isolation of radial polyalkanes are challenging due to the need to pass linear polymers through several cyclic compounds (formation of poly(pseudo)rotaxanes) and subsequent chaining.^[^ ^116c^ ^]^ Therefore, there are few reports about polycatenane‐based nanoplatforms, and we just give a few examples to illustrate the synthetic methods and application prospects of polyalkanes in the field of biomedicine.

**FIGURE 7:**
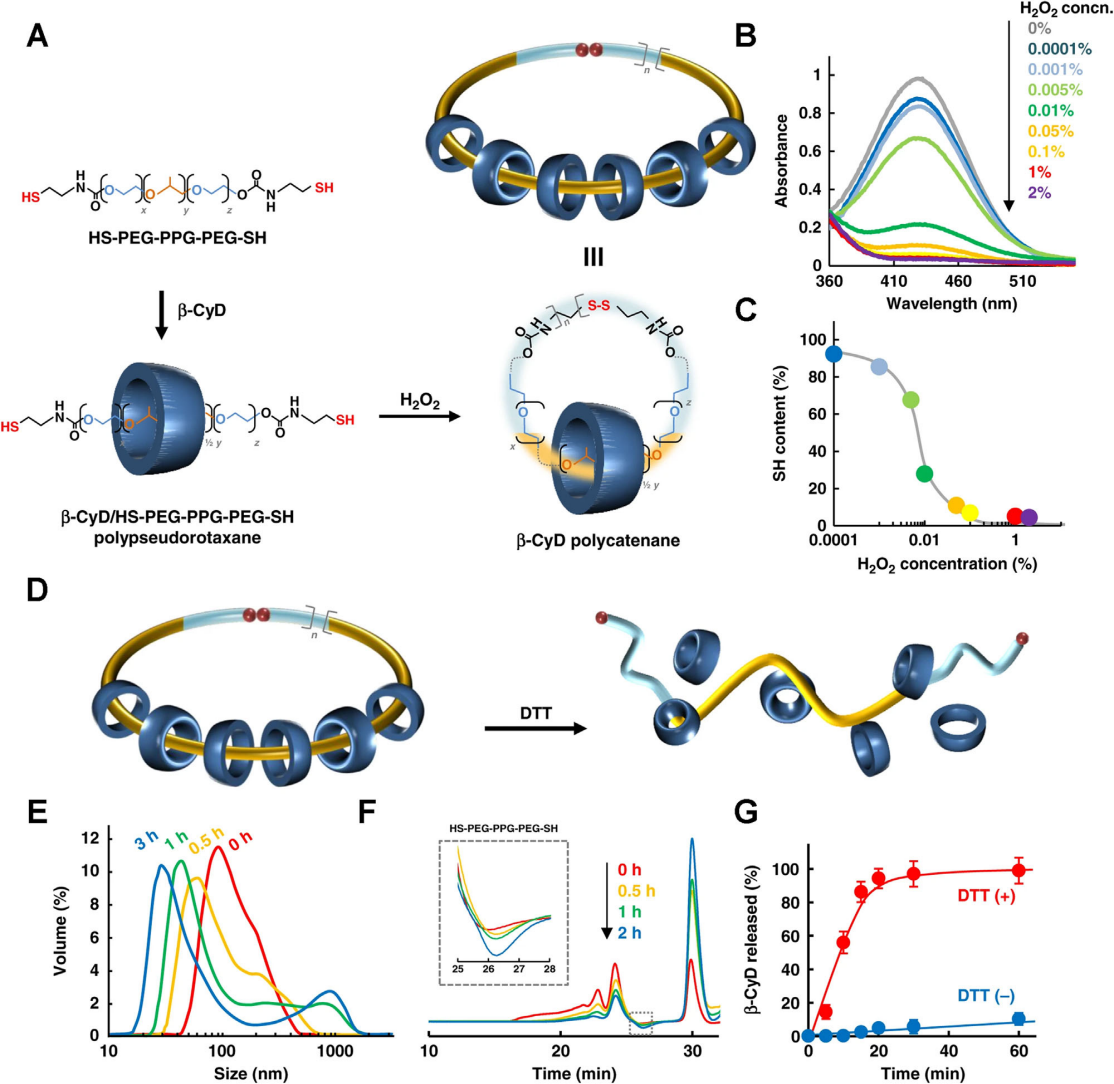
Polycatenane‐based nanocarriers. A) Synthesis and preparation of cyclodextrin (CD)‐based polycatenane. B) Absorbance peak change of 2‐nitro‐5‐thiobenzoic acid and C) thiol content change after treating the prepared polycatenane with H_2_O_2_. D) Schematic release of CD from polycatenane under redox environment. E) Nanoparticle size distribution, F) GPC profiles, and G) release profiles of polycatenanes before/after treatment with DTT. Reproduced with permission.^[^ ^44^ ^]^ Copyright 2019, Springer Nature.

The synthetic characterization and properties of polyalkanes were summarized in detail by the Stuart J. Rowan group.^[^ ^118^ ^]^ High dilution conditions are beneficial for cyclization, but simultaneously facilitate poly(pseudo)rotaxanes dissociating. Harada et al. found that α‐CD‐based polyalkanes were formed during the polymerization of 9‐anthracene‐terminated α‐CD/PEG polyrotaxanes, but the polyalkanes could not be separated from the polyrotaxanes due to their similar properties.^[^ ^43^ ^]^ Higashi et al. reported a simple method to synthesize and isolate radial polyalkanes consisting of more than 10 β‐CD units (Figure 7).^[^ ^44^ ^]^ During the formation of poly(pseudo)rotaxane strategically cyclized to produce polyalkane, the non‐cyclized poly(pseudo)rotaxanes dissociated in dimethyl sulfoxide further were isolated. Among them, carbonyldiimidazole and cystamine‐modified Pluronic P123 were activated to obtain PEG‐PPG‐PEG dithiol. Finally, PEG‐PPG‐PEG dithiol, as an axial molecule, could form disulfide bonds under oxidative conditions, thereby cyclizing to produce polyalkanes. The obtained polyalkane supramolecules are suitable for the development of advanced biomaterials, such as nanocarriers for tumor diagnosis and treatment.

## 4 SUPRAMOLECULE‐BASED CONTROLLED RELEASE SYSTEMS AND TARGETING SYSTEMS

CDs can be widely used as drug carrier components or drug containers for tumor treatment and diagnosis.^[^ ^2^, ^119^ ^]^ Besides, CDs can also reversibly interact with specific guests such as Azo, Fc, and BM, which can dissociate in response to different stimuli.^[^ ^25^, ^58^, ^68^ ^]^ Such responsive dissociated inclusion complexes can be used as responsive components to construct controlled release systems and targeting systems to attain specific distribution and controllable release of drugs.^[^ ^10^, ^25^, ^120^ ^]^ Herein, we will describe the application of CD‐based supramolecular systems in controlled release systems and targeting systems from the following three aspects, namely supramolecular gatekeepers, responsively supramolecular linkers, and supramolecular targeting components.

### 4.1 Cyclodextrin‐based supramolecular gatekeepers

A compelling avenue of supramolecular chemistry for drug delivery enables controlled capping of (meso)porous particles via host–guest interactions.^[^ ^68^, ^121^ ^]^ According to the structure and function of supramolecular nano‐gatekeepers, they are classified into nanovalve, nanopiston, and snap‐top nanomachine.^[^ ^25a^ ^]^ The nanovalve consists of a host molecule that surrounds the guest molecule as a stem immobilized on the porous nanoparticle. Guest molecules can slide along host molecules, which is triggered by internal/external stimuli, leading to blocking and unblocking gatekeepers on porous nanoparticles.^[^ ^56^, ^76^, ^122^ ^]^ For nanopiston, the macrocyclic host is attached to the nanoparticle surface, while the guest molecule acts as a stem and is held in the circulation cavity by host–guest recognition. In response to internal/external stimuli, guest molecules move out of the host cavity, allowing drug molecules smaller than the cavity to be released.^[^ ^25^, ^123^ ^]^ The release of larger drug molecules can only be achieved by removing the host molecule. As for the nanomachine, a macrocyclic host is surrounding a surface‐immobilized guest, the end of which contains a termination group. When the termination of the guest molecule detached, the pore opens in response to stimuli.^[^ ^25^, ^124^ ^]^

Under different stimuli, such as acidic pH, redox, light, and enzyme, the supramolecular gatekeepers detach from nanoparticles, and the drugs contained in the porous nanoparticles are released. This not only prevents premature leakage of the drug but also enables the contained drug to be released in a spatiotemporally accurate manner, which is a key advance in controlled drug release.^[^ ^19^, ^125^ ^]^ Hollow mesoporous silicon nanoparticle MSNs have favorable properties, such as large volume, ordered channels, and easy modification, and are often used as carriers for various drugs to achieve targeted and controllable delivery.^[^ ^126^ ^]^ CD‐Ada, CD‐BM, CD‐Fc, and CD‐Azo interactions can be modulated by various stimuli, making them potential candidates for MSN gatekeepers.^[^ ^25^, ^120^ ^]^ Herein, we will use MSN as a model porous nanoparticle to describe the stimulus conditions that trigger the mechanical movement of supramolecular “gatekeepers.”

**FIGURE 8:**
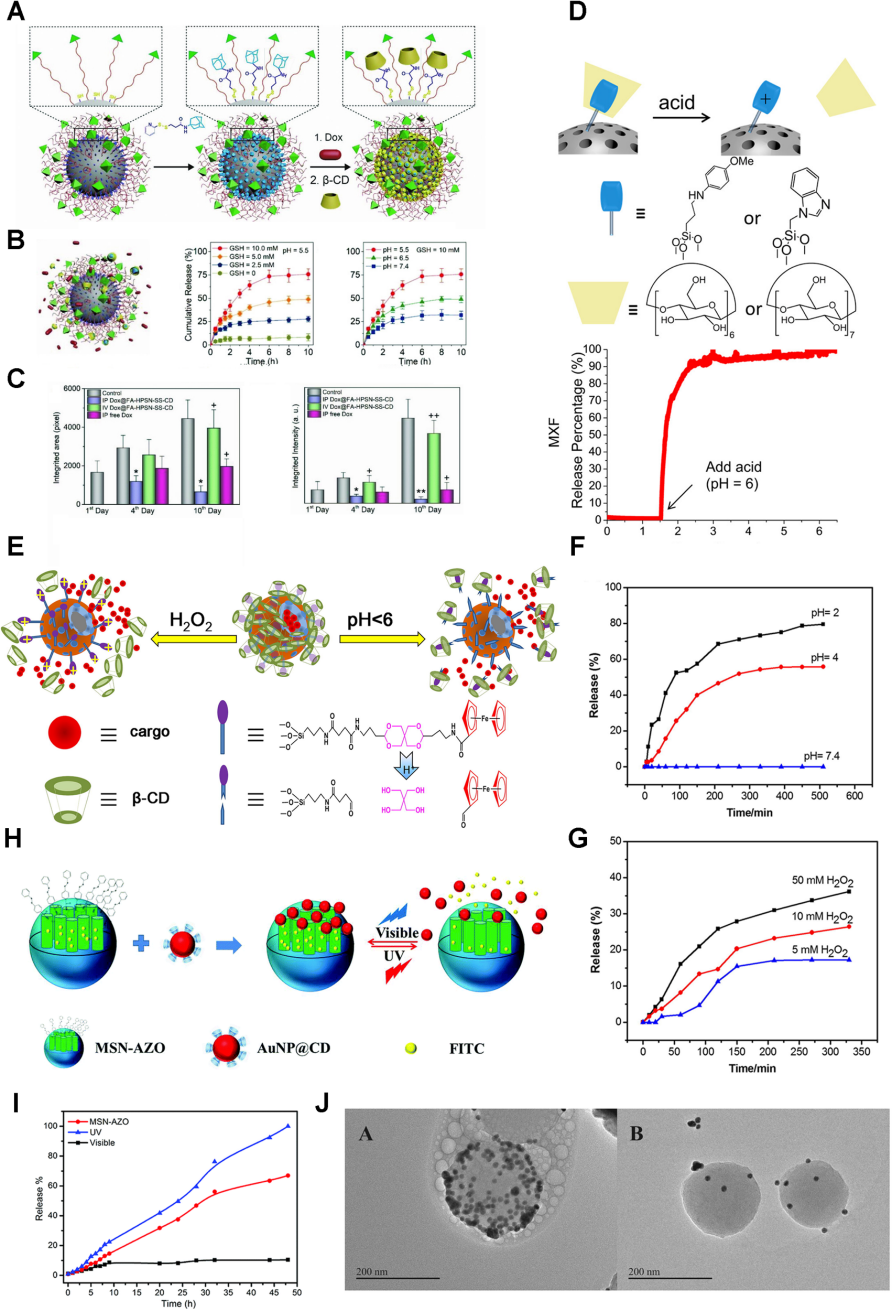
Cyclodextrin‐based supramolecular gatekeepers. A) Preparation schematics of DOX@FA‐HPSN‐SS‐CD. B) GSH‐triggered release mechanism and release profiles of DOX at different mediums. Reproduced with permission.^[^ ^45^ ^]^ Copyright 2018, Wiley‐VCH. D) Scheme of the pH‐responsive gatekeeper and MSN‐MBI‐MXF drug release profile. Reproduced with permission.^[^ ^46^ ^]^ Copyright 2015, American Chemical Society. E) Release schematics of drug molecules from HMSs‐S1. Release profiles of DOX from HMSs‐S1 at F) different pH values and G) different concentrations of H_2_O_2_. Reproduced with permission.^[^ ^47^ ^]^ Copyright 2016, Elsevier. H) Schematics of light‐responsive gatekeeper. I) Release profiles of FITC upon irradiation with different lights. J) TEM results of AuNP@CD‐capped MSN‐Azo with visible light (left) and UV irradiation (right). Reproduced with permission.^[^ ^48^ ^]^ Copyright 2018, Royal Society of Chemistry.

CD/Ada‐based supramolecular nano‐gatekeepers have been constructed recently. Zhao group grafted β‐CD/Ada groups on hollow polymer‐silica nanoparticles (HPSN) modified by folic acid (FA) via disulfide bonds and supramolecular interactions, which acted as gatekeepers to achieve the responsive release of loaded DOX (Figure 8A,B).^[^ ^45^ ^]^ The CD/Ada‐based gatekeepers were removed when disulfide bonds were broken by GSH, and then DOX was released from HPSN. Furthermore, in this study, intraperitoneal (IP) injection was employed, independent of the EPR effect. And the nanomedicines were directly applied to metastatic lesions to suppress the metastatic tumor more effectively, resulting in a better therapeutic effect (Figure 8C). Thus, CD/Ada‐based gatekeepers can be introduced on the surface of MSNs via sensitive bonds, endowing the controllable release of drugs.

Researchers have also constructed acid‐responsive MSN delivery systems based on β‐CD/BM nanovalves.^[^ ^46^, ^76^, ^123^ ^]^ Generally, they immobilized BM on the MSN surface and subsequently attached β‐CD to the BM unit using its high affinity with BM. Zink group equipped MSNs with pH‐responsive nanovalves based on β‐CD/BM interactions for the delivery of moxifloxacin (MXF).^[^ ^46^ ^]^ The gatekeepers remained closed in the physiological environment (pH 7.4) but open in an acidic microenvironment (pH < 6), releasing cargo in endosomes (Figure 8D). Briefly, the acidic environment can trigger the movement of β‐CD/BM and opening of mesopores, and the controlled release of drugs.

β‐CD/Fc‐based nano‐gatekeepers have also been constructed.^[^ ^47^, ^127^ ^]^ For example, the Wang group reported a pH and redox dual‐responsive gatekeeper based on the acid‐labile acetal group and β‐CD/Fc complex (HMSs‐S1) (Figure 8E).^[^ ^47^ ^]^ Meanwhile, they synthesized a redox‐responsive nanovalve modified with only ferrocene carboxylic acid (HMSs‐S2). HMSs‐S1 could encapsulate larger molecules including rhodamine 6G (R6G) and DOX. However, the small‐size molecule 5‐fluorouracil (5‐FU) was only loaded by HMSs‐S2. As expected, the release of DOX was facilitated in an oxidative or acid medium (Figure 8F,G). In short, the redox‐responsive gatekeepers endow MSNs with great application prospects for precise control of drug release.

Moreover, photo‐sensitive gatekeepers based on CD/Azo are extensively studied.^[^ ^48^, ^128^ ^]^ Zink group reported photoactivation of mechanized MSNs that depended on UV‐switchable guest molecules.^[^ ^128a^ ^]^ In this study, MSN was functionalized by an Azo derivative with β‐CD wrapping around the stem. After 351 nm irradiation, the Azo derivative was converted from the trans‐isomer to the cis‐isomer, leading to β‐CD detachment and drug release. Additionally, the Huizhou Liu group established a reversible light‐sensitive MSN system, consisting of Azo‐grafted MSN and CD‐modified Au nanoparticles (AuNP@CD) (Figure 8H).^[^ ^48^ ^]^ Under visible light irradiation, Azo on the MSNs exhibited a trans‐configuration and bound with AuNP@CD, so the mesopores were closed and cargoes were encapsulated. Otherwise, under UV light, trans‐Azo transformed to cis isomer, causing AuNP@CD caps to disaggregate and cargo molecules to release (Figure 8I,J). Therefore, these light‐responsive interactions between CD and Azo can switch the attachment/dissociation of caps on MSNs under lights at various wavelengths, triggering the loading/unloading of cargoes.

Additionally, the introduction of supramolecular gatekeepers through acid‐sensitive intermediate bonds enables precise control of drug release timing and dose by dual stimulation.^[^ ^47^ ^]^ Wang and co‐workers grafted β‐CD/Fc complexes on MSN surfaces via acid‐labile acetal bonds. The gatekeeper β‐CD/Fc showed excellent responsiveness to pH and H_2_O_2_ stimulation (Figure 8E–G).^[^ ^47^ ^]^ Similarly, Cheng and co‐workers constructed a pH‐ and light‐responsive MSN system modified by imine‐bonded β‐CD and Azo derivatives.^[^ ^129^ ^]^ Only when both UV light irradiation (365 nm) and acidic environment (pH = 5.0) were satisfied, would the β‐CD/Azo gatekeeper be opened and most cargoes released. Otherwise, only a few cargoes are released when given only one stimulation. Therefore, dual responsiveness makes supramolecular gatekeepers possess potential applications in designing and developing MSN systems.

In short, supramolecular nano‐gatekeepers based on CD‐Ada, CD‐BM, CD‐Fc, and CD‐Azo interactions have great potential for controllable drug release from porous nanoparticles such as MSNs. And with further rational design, dual stimulus‐responsive drug release can be achieved without much effort, which further reduces the toxic and side effects caused by non‐specific accumulation.

### 4.2 Cyclodextrin‐based responsive supramolecular linkers

It is worth noting that some drugs, such as CPT, PTX, and DOX, can be directly embedded into the host molecule and loaded into specific nanocarriers as guest molecules according to their corresponding sizes and structures.^[^ ^2^, ^17^, ^20^ ^]^ Some drugs cannot be directly encapsulated but can be loaded by introducing guest‐linked parent drugs. Therefore, these non‐covalent interactions can act as the driving force for encapsulating drugs and control the responsive release of drugs under specific stimuli. This strategy avoids premature leakage during circulation, thereby increasing drug accumulation at tumor sites and reducing toxicity to healthy organs/tissues.

**FIGURE 9:**
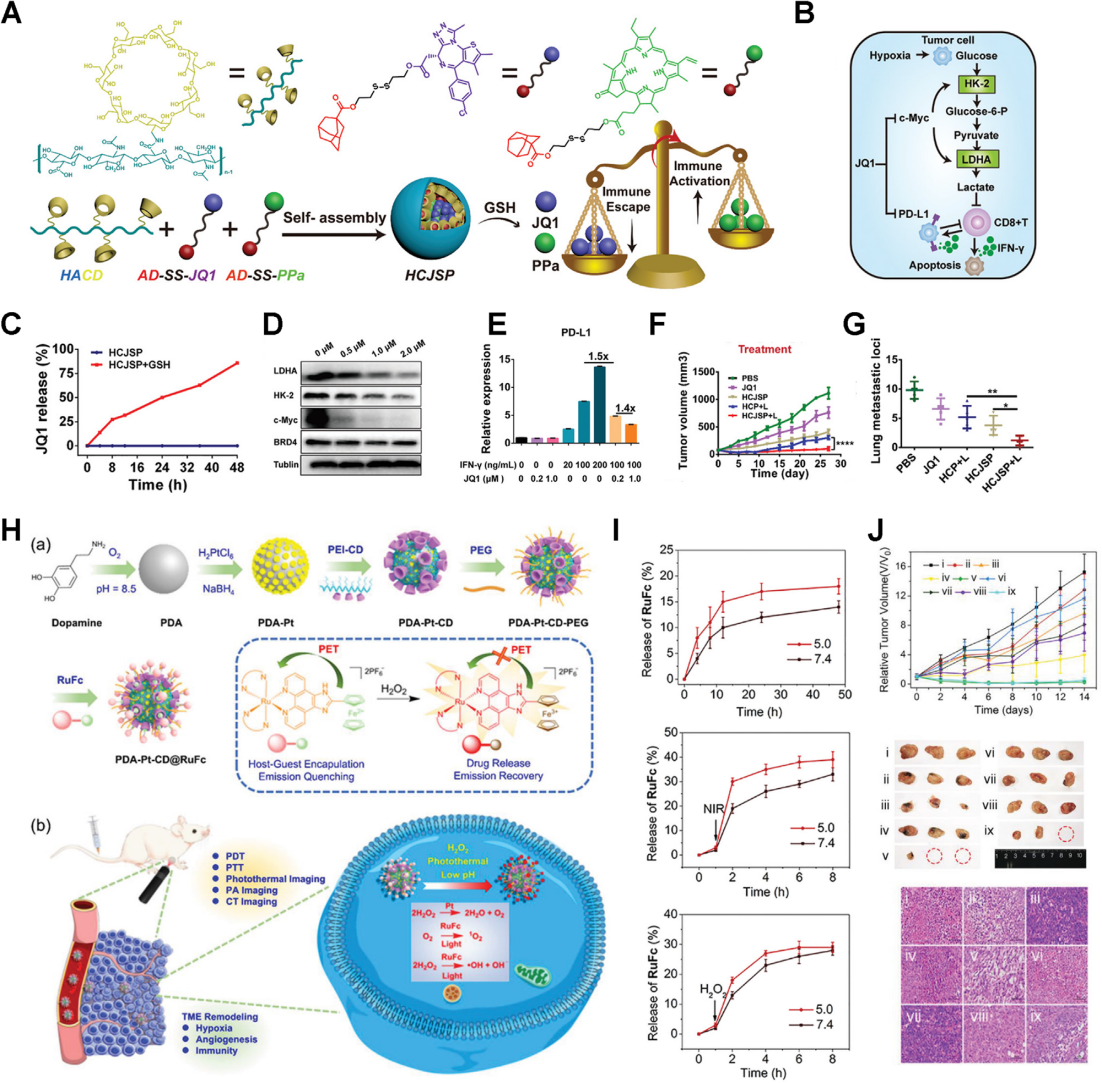
Cyclodextrin‐based responsive supramolecular linkers. Schematics of A) HCJSP nanoparticle preparation and B) combinational therapy mechanism. C) Release profiles of JQ1 from HCJSP nanoparticles. D) Western blot assay of LDHA, c‐Myc, HK‐2, and BRD4 expression after JQ1 treatment. E) Flow cytometry analysis of PD‐L1 expression after stimulation of IFN‐γ/JQ1. F) Antitumor effect and G) anti‐metastasis effect of various formulations in vivo. Reproduced with permission.^[^ ^49^ ^]^ Copyright 2021, Wiley‐VCH. H) Schematics of (a) fabrication of PDA‐Pt‐CD@RuFc NPs and (b) expected mechanisms of PDA‐Pt‐CD@RuFc NPs. I) Release profiles under different stimuli. J) Antitumor efficiency of PDA‐Pt‐CD@RuFc NPs in vivo. Reproduced with permission.^[^ ^50^ ^]^ Copyright 2020, Wiley‐VCH.

Similarly, CD‐Ada/AD, CD‐BM, CD‐Fc, and CD‐Azo inclusion complexes can also act as sensitive linkers to load drugs and control the release of drugs from supramolecular nanoplatforms. As reported, β‐CD was grafted on HA to obtain HA‐CD, which bound to adamplatin prodrug through host–guest interaction, and finally self‐assembled to SNPs.^[^ ^130^ ^]^ Furthermore, Haijun Yu and co‐workers conjugated photosensitizer (pyropheophorbide a, PPa) and epigenetic drug (JQ1) with AD via GSH‐responsive disulfide linkages to obtain AD‐SS‐PPa and AD‐SS‐JQ1, respectively, which interacted with HA‐CD to form supramolecular prodrugs (Figure 9A–G).^[^ ^49^ ^]^ The release of PPa and JQ1 was triggered by GSH, after the accumulation of nanomedicines in tumor cells (Figure 9C). JQ1 inhibited immunosuppressive components including c‐Myc and PD‐L1 expressing, combing with PDT to inhibit the progression and metastasis of pancreatic cancer (Figure 9B,D–G). As for CD/Fc linker, Mao et al. reported a theranostic nanoplatform consisting of β‐CD polydopamine‐platinum nanoparticle (PDA‐PtCD) and Fc‐attached ruthenium (RuFc) (Figure 9H).^[^ ^50^ ^]^ After accumulation in the tumor site, RuFc releasing from the nanocomposites could be induced by acid, hyperpyrexia, and ROS, combining photothermal therapy and PDT (Figure 9I,J). Therefore, the supramolecular interactions can also act as responsive linkers to introduce drugs and develop controlled release systems.

### 4.3 Cyclodextrin‐based supramolecular targeting nano‐systems

The introduction of targeting units is a viable strategy to improve tumor targeting, and supramolecular design offers many interesting avenues for the development of targeting systems. Supramolecular systems can be modified with targeting units such as RGD cyclic peptides, biotin, folic acid, and transferrin, to deliver therapeutic payloads to desired target sites.^[^ ^10^, ^19^, ^51^, ^131^ ^]^ By controlling the component ratios, the density of targeting units can be controlled. And it is even possible to present multiple targeting units on a single particle to facilitate combinatorial targeting.^[^ ^56a^ ^]^ By rationally designing poly(pseudo)rotaxane complexes, precise control of the valence and spacing of targeting groups on nanomedicines can also be achieved.^[^ ^132^ ^]^

**FIGURE 10:**
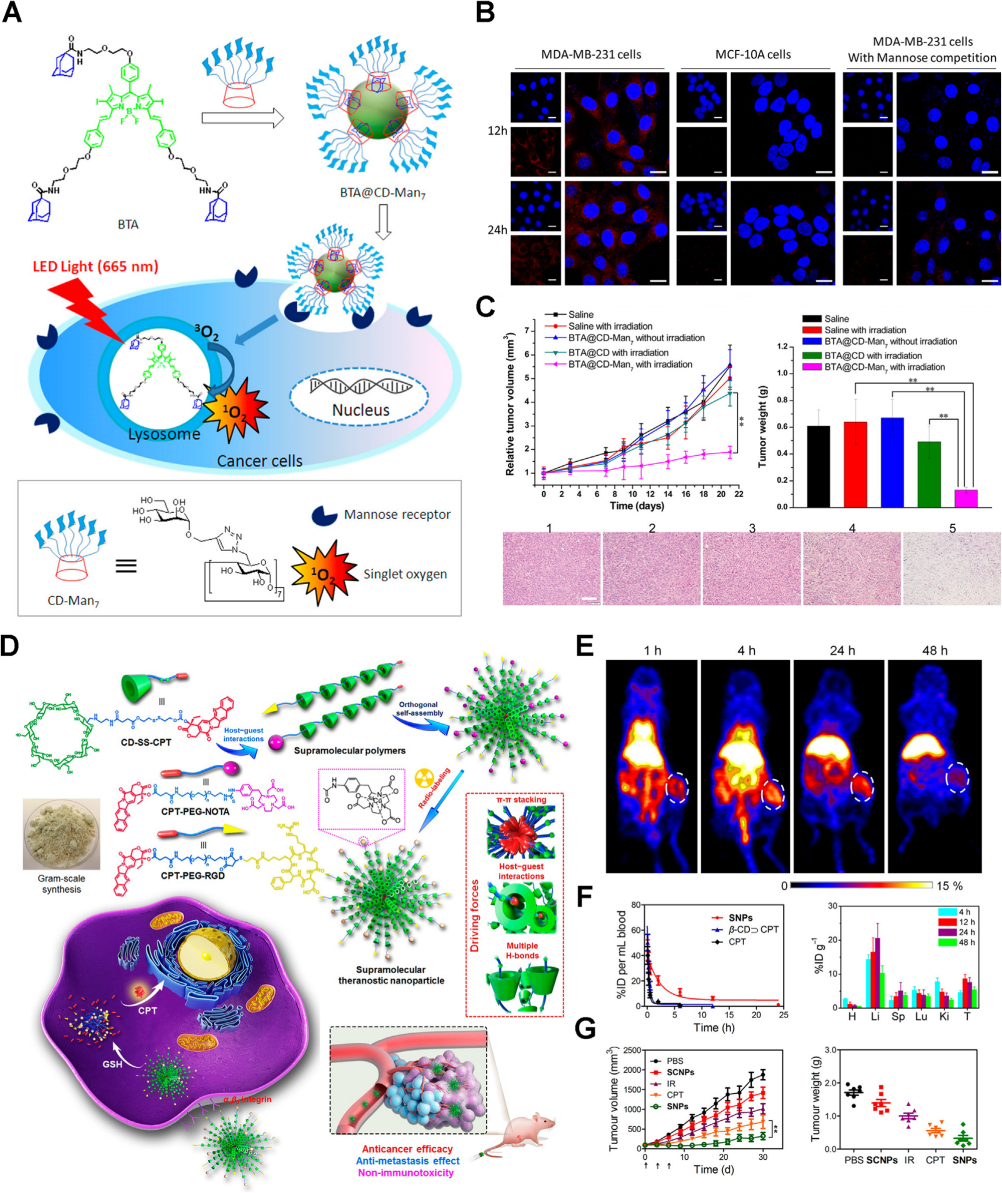
Cyclodextrin‐based supramolecular targeting nano‐systems. A) Construction schematics of BTA@CD‐Man_7_ nanoparticles and mechanisms of targeting PDT. B) The targeting efficiency of BTA@CD‐Man_7_ nanoparticles in vitro. C) The improved antitumor efficiency of BTA@CD‐Man_7_ nanoparticles through targeted PDT. Reproduced with permission.^[^ ^52^ ^]^ Copyright 2016, American Chemical Society. D) Schematic illustration for the preparation of supramolecular nanoparticles (SNPs) and their application for targeted chemotherapy. E) PET imaging of ^64^Cu@SNPs in tumor‐bearing mice. F) In vivo pharmacokinetic profiles (left) and distribution of CPT content in major organs (right) post‐injection of SNPs. G) Enhanced chemotherapy of SNPs in vivo. Reproduced with permission.^[^ ^16b^ ^]^ Copyright 2018, American Chemical Society.

Some researchers use the specific recognitions between β‐CD and adamantane (AD) to introduce targeting ligands to promote specificity against cancer cell lines.^[^ ^133^ ^]^ They first grafted β‐CD and AD onto PAA to obtain PAA‐AD and PAA‐CD, respectively, and subsequently prepared AD‐modified fluorescein isothiocyanate (FICT‐AD) and folic acid (FA‐CD). The strong AD/β‐CD interactions drove the self‐assembly of SNPs. And then DOX was loaded during the self‐assembly process to realize the targeted diagnosis and treatment of breast cancer. Similarly, transferrin (Tf) can also be introduced into SNPs via β‐CD/AD inclusion.^[^ ^51^ ^]^ To improve PDT efficiency, Yin and colleagues developed a targeting nano‐system of heptamannosylated β‐CD and AD‐modified photosensitizer (BODIPY) (Figure 10A).^[^ ^52^ ^]^ These nanoparticles could target tumor cells overexpressing mannose receptors to achieve targeting PDT and mitigation of toxic side effects (Figure 10B,C). RGD cyclic peptides can also be introduced in nanomedicines by host–guest interactions. Chen et al. reported a targeted SNP in which CD‐SS‐CPT acted as a monomer while CPT‐PEG‐RGD or CPT‐PEG‐NOTA was applied as the initiator of polymerization(Figure 10D).^[^ ^16b^ ^]^ Compared to free drug and β‐CD‐CPT, the SNPs group showed more drug accumulation and exhibited better antitumor efficiency, benefitting from RGD‐mediated targeting (Figure 10E–G). Moreover, cyclic peptides (cRGDfk) can serve as terminators for poly(pseudo)rotaxane, endowing nanomedicines with excellent targeting ability.^[^ ^2b^ ^]^ For example, Chen's group prepared poly(pseudo)rotaxane nanoparticles with an amphiphilic copolymer as the axis and β‐CD containing a primary amino group as the wheel to effectively load anticancer drugs. The cRGDfk acted as poly(pseudo)rotaxane terminators to selectively deliver drugs to tumor cells.^[^ ^134^ ^]^ In short, targeting units can be introduced into drug delivery systems via the interactions between CDs and guest molecules, such as AD, and PTX.

## 5 CONCLUSION AND PERSPECTIVE

In conclusion, this review focuses on the design progress of CD‐based nanomedicines and their potential applications in tumor diagnosis and treatment, including supramolecular nanocarriers and supramolecular attachments of nanoparticles. Through these reversible interactions, CDs can not only form poly(pseudo)rotaxanes and polyalkanes with polymers, or combine the hydrophobic and hydrophilic domains of polymers, and further self‐assemble to supramolecular nanocarriers with high loading capacity, but also be cross‐linked with polymers to self‐assemble into hydrogels or networks for continuous release of loaded drugs at the tumor site. Moreover, CDs form reversible inclusion complexes with some specific small‐molecule guests, such as BM, Azo, Fc, and Ada/AD, which dissociate in response to different stimuli. Hence, host–guest complexes can serve as nanoparticle attachments, such as supramolecular gatekeepers, responsive linkers, and targeting components, endowing nanoplatforms with remarkable functions such as controlled drug release and targeted distribution. In brief, CD‐based inclusion complexes have great broad application prospects in the field of targeted drug delivery and controlled release.

As we all know, the difference between physiological and TME is a double‐edged sword for tumor progression and the intelligent release of nanomedicines. On the one hand, nanodrugs can be passively accumulated in tumors mediated by the EPR effect, and the additional introduction of targeting groups can further improve tumor targeting efficiency. Moreover, compared with the physiological environment, the TME possesses some striking features,^[^ ^135^ ^]^ such as slight acidity, high levels of ROS, hypoxia, and highly expressed enzymes including hyaluronidase, MMPs, and furin, which also provide ideas and opportunities for the design of controllable nanomedicines.^[^ ^136^ ^]^ On the other hand, it is now clear that the distribution of nanomedicines facilitated by the EPR effect is limited resulting from the highly complex TME, including high interstitial fluid pressure and elevated solid stress.^[^ ^137^ ^]^ Meanwhile, cancer cells can downregulate tumor‐associated antigens on the surface, induce the expression of immunosuppressive molecules, and an inherent immunosuppressive microenvironment is formed, thereby achieving immune escape.^[^ ^136b^ ^]^ Therefore, there is an urgent need to construct intelligent nanomedicines to overcome the above problems and improve antitumor efficiency.

Nanomedicines with transformable shapes and sizes, dual responsiveness, programmable targeting capacity, or combination therapy have great potential to solve the above problems.^[^ ^138^ ^]^ For example, nanoplatforms with stimuli‐responsive increased size can accumulate in the tumors through the EPR effect and active targeting, and then retain in the tumor sites, benefiting from the stimuli‐responsive shape transformation or aggregation.^[^ ^23^, ^139^ ^]^ On the other hand, the stimuli‐sensitive size‐shrinkable nanomedicines can accumulate in tumors and penetrate the deep sites of tumors, due to the decreased size.^[^ ^23^, ^140^ ^]^ More and more reports have verified that CD‐based supramolecules can play a vitally important role in constructing smart nanomedicines to motivate a better antitumor effect, which can realize enhanced retention and penetration via changeable shape and size, dual responsiveness, programmable targeting capacity, or combination therapy.^[^ ^30^, ^34^, ^37^ ^]^ Additionally, some CDs have pharmacological activities of their own, for example, β‐CD can interact with cholesterol, being applied for the treatment of atherosclerosis or the detection of cholesterol.^[^ ^141^ ^]^ Therefore, CDs have broad application prospects in the field of drug delivery and tumor diagnostics.

Briefly, with high stability, excellent biocompatibility, and easy modification, CDs can encapsulate guest molecules of suitable size with different binding affinities. Through host–guest interactions, nano‐framework, hydrophobic drugs, response components, and targeting ligands can be introduced into supramolecular nanodrugs to achieve various goals for tumor therapy, such as controllable drug release and targeted delivery. Additionally, CD‐based supramolecules can be utilized to achieve shape or size transformation,^[^ ^34^, ^142^ ^]^ programmable targeting, and combination therapy, facilitating the treatment and diagnosis of cancers. Therefore, CD‐based smart supramolecular nanomedicines may provide diverse strategies for precise cancer diagnosis and therapy.

## CONFLICT OF INTEREST STATEMENT

The authors declare no conflict of interest.
